# Relevant Condition at Death and customized birthweight centiles for stillbirth classification: A retrospective cohort study

**DOI:** 10.5339/qmj.2025.71

**Published:** 2025-08-17

**Authors:** Azra Kazmi, Devi Krishna, Amr Abdel Aziz, Malika Boumedjane, Faten El Taher, Victor Olagundoye, Fathima Minisha, Thomas Farrell

**Affiliations:** 1 Department of Obstetrics and Gynecology, Women’s Wellness and Research Center, Hamad Medical Corporation, Doha, Qatar; 2 Department of Research, Women’s Wellness and Research Center, Hamad Medical Corporation, Doha, Qatar; 3 College of Medicine, Qatar University, Doha, Qatar *Email: akazmi@hamad.qa

**Keywords:** Customized GROW chart, fetal growth restriction, Qatar, ReCoDe, SGA (small for gestation age), stillbirth

## Abstract

**Background:**

Stillbirths (SBs) remain the largest contributor to perinatal mortality, with nearly two-thirds of SBs consistently reported as unexplained. Studies have consistently shown fetal growth restriction (FGR) as a major contributor to SB. Early detection and appropriate management are vital to reducing SB, and the Relevant Condition at Death (ReCoDe) classification utilizes customized birthweight (CBW) centiles to diagnose FGR, decreasing the proportion of SB remaining unclassified.

**Methods:**

This was a retrospective cohort study to classify SBs using the ReCoDe classification system. A random sample of SBs above 24 weeks’ gestational age was included, and relevant clinical, laboratory, and histopathological diagnoses were extracted from medical records. The birthweights were customized according to maternal height, weight, ethnicity, parity, fetal biological sex, andgestational age at diagnosis of SB to obtain CBW centiles. FGR was defined as CBW centiles <10 percentile.

**Results:**

The mean age of the 254 women included was 30.5 (standard deviation = 6.2) years, with 17% being <25 years and 9.1% being ≥40 years. The majority were multiparous, 12% grand multiparous. The mean body mass index at booking was 28.6 ± 5.6 kg/m2, with 31% being in the obese category (?30 kg/m2). FGR was the most common diagnosis (52%), followed by maternal diabetes (24.0%), placental abruption (16.5%), other major placental insufficiency (15.4%), lethal congenital anomalies (13.8%), and chorioamnionitis (13.8%). The most common primary diagnosis was FGR (37.0%), followed by congenital anomalies (13.8%), placental abruption (9.1%), and maternal diabetes (6.7%). FGR remained the leading primary diagnosis in non-anomalous babies (43%), with >63% with a secondary diagnosis. The most common secondary diagnosis associated with FGR was other placental insufficiency (23.4%), followed by abruption and maternal conditions. After applying the classification system, only 18 cases remained unclassified (7.1%).

**Conclusions:**

Applying the ReCoDe classification with CBW centiles, we were able to classify most of our SBs, with only 7.1% remaining unclassified. Appropriate classification of SB is vital for understanding what went wrong, counselling bereaved families, planning future pregnancies, and improving perinatal care. Early identification of FGR will allow appropriate monitoring and timely delivery.

## INTRODUCTION

Stillbirth (SB) is one of the most traumatic experiences for couples, families, and healthcare providers. Despite over 2 million SBs occurring annually worldwide and many classification systems in use around the world, SB reporting remains inconsistent, with over two-thirds of cases reported as unexplained or nonspecific.^1^ Studies have shown that a significant number of SBs occur at gestational age (GA) when the babies would have survived if delivered in good condition and, in many cases, are preventable.^2^ Therefore, the SB rate can be seen as a good indicator of the quality of maternity care.

Since SB is often multifactorial, an effective classification system for SBs is crucial to understanding and identifying what went wrong, which is vital to improving clinical practice. The first classification system for the audit and surveillance of SB was proposed in 1954 by Sir Dugald Baird and his colleagues in Aberdeen, Scotland.^3^ Since then, other classification systems have been introduced, such as the Wigglesworth classification,^4^ the Tulip classification,^5^ and the International Classification of Diseases-Perinatal Mortality (ICD-PM) classification.^6^ However, most of the classifications still report a considerable number of SBs as unexplained and varied regarding the hierarchy of diagnoses.

In 2005, Gardosi et al., in an effort to reduce unclassified SB, described a new classification system that is based on identifying the relevant condition at the time of fetal death in utero (ReCoDe [Relevant Condition at Death] classification). Using the ReCoDe classification to analyze a regional cohort of 2,625 SBs, they were able to classify 85% of the SBs compared to 66% of unexplained SBs with the Wigglesworth classification.^7^ The major change was the use of customized birthweight (CBW) centiles to classify intrauterine fetal growth restriction (FGR), which dramatically improved the classification.^8^

The ReCoDe classification system is one of the few frameworks created specifically to determine the causes of SB, focusing on what went wrong rather than the reason behind it. It is structured hierarchically, beginning with conditions affecting the fetus and progressing outward in an anatomical manner, categorizing them into fundamental anatomical groups, which are then subdivided into pathophysiologic conditions, with the most pertinent condition for a case listed first. This novel methodology allows researchers and healthcare providers to effectively analyze existing databases without requiring thorough investigations of each case. A notable feature of the ReCoDe classification is its clear hierarchical structure, which is firmly based on International Classification of Diseases codes, enhancing its practicality and efficacy. Impressively, it enables the identification of a relevant condition in approximately 85% of SB cases. Of particular importance is the strong focus on FGR within the ReCoDe framework. This emphasis aligns with and supports earlier findings regarding the underlying pathophysiology of SBs, highlighting the significant opportunity for preventing SBs associated with FGR. Nevertheless, this focus may inadvertently minimize essential insights into other related conditions. Autopsies and examinations of the placenta can provide vital information about placental pathology, which often precedes both FGR and SB. These insights are critical for understanding a wider range of clinical scenarios related to maternal vascular disease and FGR. In conclusion, while the ReCoDe classification serves as a powerful resource for comprehending SBs, it is crucial to take into account the complete clinical context to ensure that no important information is neglected.^9^

A study by Maducolil et al. comparing different classification systems on a cohort of 120 cases of SB in Qatar reported ReCoDe and ICD-PM classifications as superior in providing explanations for SBs, with unexplained causes around 10% for each group.^10^

In Qatar, a standardized classification system to determine and qualify SBs is still lacking, and there exists a deficiency in obtaining consistent information about the nature and cause of SBs, essential for planning health provision, improving maternity standards, and providing appropriate counselling to grieving mothers and families.

In this study, we report the maternal demographics associated with a representative cohort of SBs in Qatar and classify the associated clinical and histopathological diagnoses based on the ReCoDe system, aiming to reduce the proportion remaining unclassified. Additionally, we use CBW centiles to define FGR to evaluate its impact on SB classification compared to the population- based definition of FGR. We chose the ReCoDe classification system as it is simple to use and relies mainly on clinical and laboratory information, which is important in places like Qatar, where postmortem examination of stillborn fetuses is very rare.

## MATERIALS AND METHODS

### Study design and setting

This was a retrospective single-center observational study performed at Women’s Wellness and Research Center (WWRC)—the largest teaching hospital in Qatar, with over 18,000 deliveries per annum, accounting for approximately 70% of the deliveries in the country. Therefore, our study does not represent all the SBs in the country.

Approval for the study was granted by the Hamad Medical Corporation (HMC) institutional review board (number: MRC-01-22-356) before conducting the study, with waiver of informed consent as only preexisting data from patient medical health records were used for the analysis. The research was conducted in full conformance with the principles of Good Clinical Practice and within the laws and regulations of the Ministry of Public Health in Qatar.

### Participants

The study included a random selection of women attending the WWRC with a diagnosis of SB between 2017 and 2021. SB was defined as a baby born after 24 weeks’ GA with no signs of life at the time of birth. We have used 24 weeks, as that is the cutoff for viability in our hospital. It is still the recommended cutoff gestation by the World Health Organization and many other parts of the world. We have not classified the SBs into early, late, and term as we have not used the classification in our analysis. The SB rate in WWRC is 6.6 per 1,000 total births as per internal intrauterine fetal death (IUFD) committee reports, which translates to approximately 112 SB in the hospital per year (approximately 17,000 births per year). We randomly selected at least 50 cases per year to get a representative sample size of 254 SB over 5 years. All women with SB were eligible for inclusion in the study. We arranged the cases according to the delivery date and then randomly selected 50 cases per year using a random number generator to give a total of 254. FGR based on customized centiles was one of our most relevant classifications, as customization is still not practiced in Qatar, and we were aiming to highlight its importance in the classification of SBs. Based on prior studies, 43% of the SBs were growth-restricted based on customized centiles. In order to be able to detect this similar prevalence in our population, at α = 0.05 and an error margin of 5%, we would need approximately 225 cases of SBs. Considering this as well, we decided to use at least 50 per year, randomly selected.

Inclusion criteria were babies born with no sign of life at or after 24 weeks of gestation age from 2017 to 2021, while babies born with no sign of life before 24 completed weeks of gestation during the study period were excluded.

### Data source and variables

After sample selection, the hospital record number was used to access the maternal electronic health records to extract the required data. The charts were retrieved using ICD10 coding (p95) for IUFD or SB, and only pregnancies above 24 weeks’ GA were included. Descriptive variables collected included age in completed years, gravidity-defined as the number of previous pregnancies, parity-defined as previous pregnancies above 24 weeks GA (divided into nulliparous, multiparous 1–4 deliveries and grand multiparous >4), nationality (Qatari and non-Qatari), maternal booking height, weight and body mass index (BMI), GA at the time of SB diagnosis and if the diagnosis was during the antenatal period or intrapartum, GA at time of birth, mode of delivery (vaginal vs. cesarean section), fetal biological sex and birthweight in grams.

The ReCoDe classification system has a hierarchy of possible causes for SB.^7^ The sequence starts from fetal causes (group A), progressing anatomically outward to include cord-related, placental, amniotic fluid-related, uterine, maternal, intrapartum, and finally unclassified (group I), with each group having subdivisions resulting in 36 diagnoses. The birthweights were customized based on maternal nationality, height, weight, parity, GA at diagnosis of SB, and fetal biological sex,^8^ to generate CBW centiles using the Gestation-Related Optimal Weight (GROW) application. FGR was defined as CBW centiles <10th percentile on the GROW charts. All diagnoses were determined based on clinical examination, laboratory results, histopathological findings, or physician documentation. The primary and secondary diagnoses were the first and second diagnoses assigned to each case in the medical records, according to the ReCoDe hierarchy. Although a third and fourth diagnosis was possible, significance was assigned to the first two.

### Statistical analysis

Among the demographic variables, continuous data were represented as mean and standard deviation (SD) or median and interquartile range (IQR), depending on the distribution of the data. The distribution was evaluated based on histograms and the Shapiro-Wilk test. Categorical variables were represented as frequencies and percentages. The number of women having each of the diagnoses as per ReCoDe was reported as a percentage of the total number in the cohort. The frequency and percentage of women having the diagnoses as primary or secondary were calculated to develop the classification matrix.

**Figure 1.**
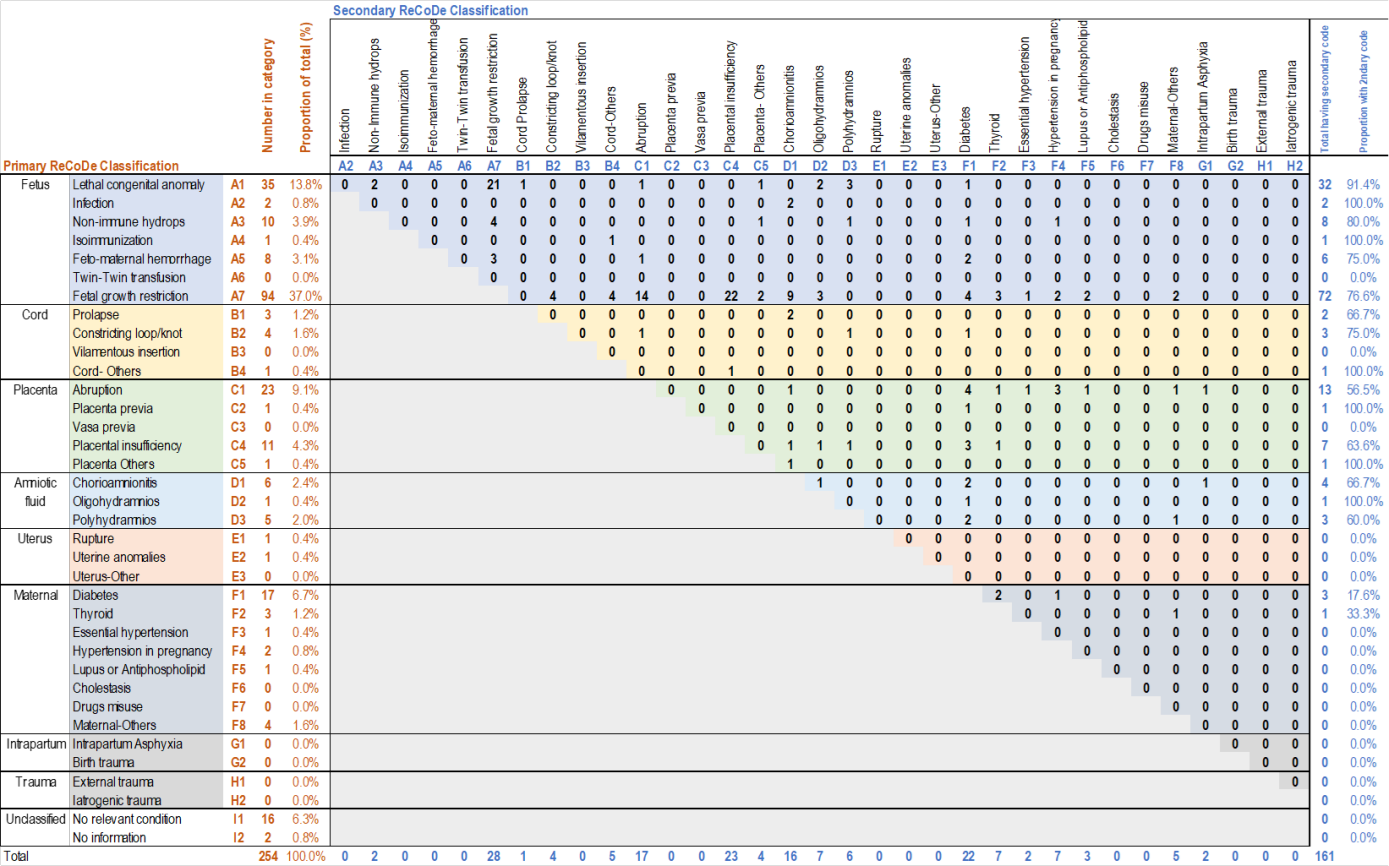
Classification matrix of stillbirths according to the Relevant Condition at Death (ReCoDe) classification. Orange numbers represent the primary diagnosis (first diagnosis as per the order mentioned). Blue numbers represent the secondary diagnosis in those with a primary diagnosis.

The ReCoDe classification matrix was developed according to the format presented by Gardosi et al.,^7^ as shown in Figure 1. The rows represented the diagnoses in the recommended order of priority from A to I, with subclassifications. The number of women and the proportion of the total having each diagnosis as the primary were represented first in each row. The columns represented the same sequence of diagnoses, and each cell represented the number of women having a secondary diagnosis among those having a primary diagnosis, with numbers at the right-hand end representing total numbers and proportions. The descriptive analysis was done in Stata Statistical Software, Release 18,^11^ and the classification matrix was developed in Microsoft Excel.

## RESULTS

The mean age of the 254 women included was 30.5 (SD = 6.2) years, with 17% being <25 years (2% teenage pregnancies), and 24% were >35 years (9.1%, >40 years). The majority were multiparous, with one-third being nulliparous and 12% grand multiparous. Seventy-five percent of women (n = 191) were non-Qatari, which is in keeping with the general demography of the country. The mean BMI at booking was 28.6 ± 5.6 kg/m2, with 31% being in the obese category (>30 kg/m2) and 2% being underweight (<18.5 kg/m2). The mean GA at diagnosis (weeks) was 32.6 ± 4.9 weeks and at delivery was 33.1 ± 4.8 weeks, with 88% diagnosed antenatally. 82% delivered vaginally, and the mean birth weight was 1,801 ± 939 g, as shown in Table 1.

Overall, FGR was the most common diagnosis (52.0%), followed by maternal diabetes (24.0%), placental abruption (16.5%), other major placental insufficiency (15.4%), lethal congenital anomalies (13.8%), and chorioamnionitis (13.8%). Nearly 26% of the cases had “no relevant condition” or “no information” documented in the records (Table 2). There were no cases with the diagnosis of twin-twin transfusion, velamentous insertion, vasa previa, drug misuse, or birth, external, or iatrogenic trauma. All diagnoses of lethal anomalies, fetal infection, isoimmunization, feto-maternal hemorrhage, and uterine anomalies were primary.

The most common primary diagnosis was FGR (37.0%), followed by congenital anomalies (13.8%), placental abruption (9.1%), and maternal diabetes (6.7%). FGR remained the leading primary diagnosis in non-anomalous babies (43%), followed by abruption (10.5%) and diabetes (7.8%). More than 63% (n = 161) had a secondary diagnosis. The most common secondary diagnosis associated with FGR as the primary was other placental insufficiency (histological evidence: C4; 23.4%), followed by abruption and maternal conditions. Thirty-seven percent of women (*n* = 94) had additional third and/or fourth diagnoses, more commonly including diagnoses down the hierarchy. Notably, 48% of the maternal conditions were neither primary nor secondary. After applying the classification system, only 18 cases remained unclassified (7.1%).

## DISCUSSION

The study classified a random sample of SBs in Qatar according to the ReCoDe classification system, aiming to reduce the number of unexplained cases. FGR was noted in 52% of the cases when FGR was defined using CBW centiles. After applying the classification, 7.1% remained unclassified (a 73% reduction from the original 26%).

SB remains one of the most traumatic and agonizing experiences for couples and families, with significant and long-lasting negative impacts on social and mental health.^12^ Appropriate classification of SB is crucial to understanding what went wrong to improve maternity standards provide counselling to grieving families, and offer an outlook for future pregnancy outcomes while ensuring healthcare resources are prioritized. Since the first classification of SB was introduced in 1954, over 30 classification systems^13^ have been developed and used worldwide, highlighting the continued efforts to reduce the unacceptably high cases of unexplained SB reported with these classifications.

Many countries, including Qatar, have no standardized classification system for SB. In this study, we have used the ReCoDe classification as it is simple to apply, significantly reduces the number of cases reported as unexplained, and relies mainly on clinical and laboratory information, which is important as postmortem examination for stillborn fetuses is very rare in Qatar. The ReCoDe is a hierarchical system of classification that starts with conditions that affect the fetus and moves outwards in anatomical groups, which are subdivided into pathophysiological conditions and further into primary and secondary conditions, with the last group being unclassified.^7^ Although ReCoDe is based on what went wrong at the time of SB rather than the underlying cause or the earliest variables in the causal pathway, the hierarchy of diagnoses makes it easier to provide answers to grieving parents.

Using the ReCoDe classification system, we were able to identify relevant conditions at death in utero for nearly 93% of the cases and reduced the unexplained SBs from 26% to 7.1%. FGR was the most common condition associated with SBs in our study, accounting for 52% of the cases, and the placental insult is evidenced by at least 77% of these SBs having a secondary diagnosis, including abruption and histopathological placental insufficiency. A previous study exploring SB in Qatar reported FGR in only 22.5% based on Hadlock ultrasound growth charts.^10^ Using CBW centiles, we were able to identify more than double the proportion of FGR among the SBs in the country. This supports previous studies that directly compared Hadlock with customized charts in SBs, showing a much better correlation with the GROW charts.^14^ While FGR is not exactly a cause of death, it is an important factor associated with SB.^15^ Our study reaffirms this association and notably reports FGR as a primary diagnosis in 94 non-anomalous babies (37% of the total).

Studies have shown that a significant number of SBs are due to substandard care and, therefore, are preventable. Gardosi et al. highlighted the failure of healthcare professionals in identifying babies that are pathologically small and planning timely delivery before fetal death as a major contributor to substandard care.^7^ They stressed the limitations of the current widely used population growth charts to differentiate between babies that are small-normal with no associated increased risk of adverse outcome and babies that are pathologically small with increased risk of perinatal morbidity and mortality. An analysis of maternity data from over 2.2 million pregnancies compared three population- based growth charts (Hadlock, Intergrowth-21, and World Health Organization) with GROW charts and reported the superiority of GROW in identifying more pregnancies with FGR.^14^ Studies from the US report the much higher associations of SB with birthweights that are individualized, over population-based definitions of FGR.^16^ Other studies have reported that small for gestational age based on GROW charts is a better predictor of pregnancy complications and adverse outcomes, including SBs and neonatal deaths, than population-based growth charts.^17–19^

This study also reports maternal demographic details associated with SBs. In Qatar, the mean maternal age is 30.2 years, with 2% teenage pregnancies, similar to our study.^20^ However, the mothers in our cohort were 9% likely to be above 40 years, which is higher than the 5% in the general population. Similarly, the mean BMI in the general population is 29.5 kg/m^2^; however, 30% of our cohort were in the obese category compared to 41% in the general population. Notably, 2.1% of our cohort were underweight compared to only 0.3% in the general population.^20^ We also report a much higher proportion of grand multiparity in our cohort at 12.2% compared to 6.6% in the population. This is similar to previous studies that report that mothers having SBs are more likely to be either older, underweight, or grand multiparous.^21,22^ We found that by applying the GROW chart to SBs in our study, we were able to identify more babies that are growth-restricted and significantly reduce the number of unexplained SBs by 73% (from 26% to 7.1%). The previous study on SBs from Qatar compared different classification systems and reported that 12% remained unclassified using ReCoDe, which is higher than ours, most likely due to the differences in the classification of FGR.^10^

That study also reported that ReCoDe and ICD- PM were the best classification systems for Qatar. However, the proportion unclassified was the best in our study when combining ReCoDe with the GROW definition of FGR. Early identification and diagnosis of growth-restricted babies are key to instituting appropriate monitoring and timely delivery to reduce adverse outcomes.

### Strengths and limitations

The use of the ReCoDe classification system and customized GROW standard chart to classify the 254 SBs in our study is a particular strength due to the large number of SBs in the study. The use of routinely collected data to classify most of our SBs has shown that the ReCoDe classification and GROW effectively classify SBs in Qatar to improve perinatal care. Certain limitations must be considered when interpreting these results. Although the sample is representative, the use of retrospective data was a limitation, as it can introduce bias due to human error during data extraction. Diagnoses may be missed due to a lack of documentation or not undergoing the required laboratory or histopathological evaluation. However, there are hospital SB protocols in place that require the completion of a battery of investigations upon diagnosis, and it is unlikely that the bias associated with missed diagnoses will be significant. The investigation battery includes placental histopathology and fetal autopsy; however, fetal autopsy is infrequently performed as it is generally not embraced by the majority of parents.

## CONCLUSIONS

Using the ReCoDe classification system together with CBW centiles, we were able to classify most of our SBs, with only 7.1% remaining unclassified. An appropriate and effective classification system is vital not only to understanding what went wrong but also to providing appropriate counselling to grieving couples and planning future pregnancies. The ReCoDe classification system is simple and effective and can be adopted in Qatar. A significant number of SBs due to FGR can be prevented through the early identification of growth-restricted babies using GROW customized charts. This would allow appropriate monitoring and timely delivery.

**Acknowledgements**

The authors would like to thank the IUFD committee in WWRC for providing the sample used in this study and for the excellent ongoing work in improving the classification of SB in the country. The authors would like to thank the Medical Research Centre, HMC, for their continuous support in the conduct of this study.

**List of abbreviations**

**Table:** 

BMI	body mass index
CBW	customized birthweight
FGR	fetal growth restriction
GA	gestational age
GROW	Gestation-Related Optimal Weight
HMC	Hamad Medical Corporation
ICD-PM	International Classification of Diseases-Perinatal Mortality
IQR	interquartile range
IUFD	intrauterine fetal death
ReCoDe	Relevant Condition at Death
SB	stillbirth
SD	standard deviation
WWRC	Women’s Wellness and Research Center

**Funding**

No funding was required for the conduct of this study.

**Conflict of interest**

The authors have no conflicts of interest to declare.

## Figures and Tables

**Table 1:** Maternal and fetal demographics.

Demographics, *N* = 254		*n*		%*N*
Maternal age (mean, SD)			30.5 ± 6.17	
Maternal age	<25 years	44		17.3%
	25–34 years	150		59.1%
	≥35 years	60		23.6%
Gravidity (median, IQR)			3 (1–5)	
Parity	Nulliparous	82		32.3%
	Multiparous	141		55.5%
	Grand multiparous	31		12.2%
Nationality	Qatari	63		24.8%
	Non-Qatari	191		75.2%
Maternal height (mean, SD)			158.5 ± 6.4	
Maternal weight at booking (mean, SD)			72.0 ± 16.1	
Maternal BMI at booking (mean, SD)			28.6 ± 5.6	
GA at diagnosis of SB (days; mean, SD)			228.2 ± 34.5	
GA at diagnosis of SB (weeks; mean, SD)			32.6 ± 4.9	
GA at delivery (days; mean, SD)			231.6 ± 33.4	
GA at delivery (weeks; mean, SD)			33.1 ± 4.8	
Mode of delivery	Vaginal birth	208		81.9%
	Cesarean delivery	46		18.1%
Timing of SB	Antenatal	225		88.6%
	Intrapartum	29		11.4%
Fetal biological sex	Male	129		50.8%
	Female	115		45.3%
	Undetermined	10		3.9%
Birthweight in grams (mean, SD)			1,801 ± 939	

BMI: body mass index, GA: gestational age, IQR: interquartile range, SB: stillbirth, SD: standard deviation

**Table 2:** Number and proportion of diagnoses as per the Relevant Condition at Death classification.

Classification of diagnoses		Total *N* = 254 *n, %N*	Proportion as primary diagnosis	Proportion as secondary diagnosis
**Group A: Fetus**				
A1	Lethal congenital anomaly	35 (13.8%)	35 (100%)	
A2	Infection	2 (0.8%)	2 (100%)	0 (0%)
A3	Nonimmune hydrops	12 (4.7%)	10 (83.3%)	2 (16.7%)
A4	Isoimmunization	1 (0.4%)	1 (100%)	0 (0%)
A5	Feto-maternal hemorrhage	8 (3.2%)	8 (100%)	0 (0%)
A7	Fetal growth restriction^†^	122 (52.0%)	94 (77.0%)	28 (23.0%)
**Group B: Cord**				
B1	Cord prolapse	4 (1.6%)	3 (75.0%)	1 (25.0%)
B2	Cord constricting loop or knot	8 (3.2%)	4 (50.0%)	4 (50%)
B4	Others	6 (2.4%)	1 (16.7%)	5 (83.3%)
**Group C: Placenta**				
C1	Abruption^*^	42 (16.5%)	23 (54.8%)	17 (40.5%)
C2	Placenta previa^*^	2 (0.8%)	1 (50.0%)	0 (0.0%)
C4	Other major placental insufficiency^*^	39 (15.4%)	11 (28.2%)	23 (59.0%)
C5	Others	5 (2.0%)	1 (20.0%)	4 (80.0%)
**Group D: Amniotic fluid**				
D1	Chorioamnionitis^*^	35 (13.8%)	6 (17.1%)	16 (45.7%)
D2	Oligohydramnios^*^	19 (7.5%)	1 (5.3%)	7 (36.8%)
D3	Polyhydramnios^*^	15 (5.9%)	5 (33.3%)	6 (40.0%)
**Group E: Uterus**				
E1	Uterine rupture^*^	2 (0.8%)	1 (50.0%)	0 (0.0%)
E2	Uterine anomalies	1 (0.45)	1 (100%)	0 (0.0%)
**Group F: Maternal**				
F1	Diabetes^*^	61 (24.0%)	17 (26.6%)	22 (36.1%)
F2	Thyroid^*^	20 (7.9%)	3 (15.0%)	7 (35.0%)
F3	Essential hypertension^*^	8 (3.2%)	1 (12.5%)	2 (25.0%)
F4	Hypertension in pregnancy^*^	25 (9.8%)	2 (8.0%)	7 (28.0%)
F5	Lupus or antiphospholipid^*^	9 (3.5%)	1 (11.1%)	3 (33.3%)
F6	Cholestasis^*^	2 (0.8%)	0 (0.0%)	0 (0.0%)
F8	Others^*^	16 (6.3%)	4 (25.0%)	5 (31.3%)
**Group G: Intrapartum**				
G1	Intrapartum asphyxia^*^	4 (1.6%)	0 (0%)	2 (50.0%)
**Group I: Unclassified**				
I1	No relevant condition	54 (21.3%)	16 (29.6%)	-
I2	No information	12 (4.7%)	2 (16.7%)	-

Note: Groups A6 (twin–twin transfusion), B3 (velamentous insertion), C3 (vasa previa), E3 (other uterine factors), F7 (drug misuse), G2 (birth trauma), H1 (external trauma), and H2 (iatrogenic trauma) not shown due to nil observations.

*Remaining numbers present as third or fourth diagnosis.

^†^Fetal growth restricted calculated as <10th customized centiles.
